# Clinical Characteristics Analysis of Vestibular Migraine Combined With Unruptured Intracranial Aneurysm

**DOI:** 10.1002/brb3.70782

**Published:** 2025-08-22

**Authors:** Yaoheng Zhang, Yi Ju, Chunling Liu, Hui Li, Yanlu Jia, Shuning Sun, Haozhe Yin, Suisui Ma, Wenbo Peng

**Affiliations:** ^1^ Department of Neurology The Second Affiliated Hospital of Zhengzhou University Zhengzhou China; ^2^ Department of Neurology Beijing Tiantan Hospital Beijing China

**Keywords:** 3D‐Slicer, aneurysm rupture, intracranial aneurysm, migraine, vestibular migraine

## Abstract

**Background:**

Patients with vestibular migraine (VM) and those with migraine accompanied by unruptured intracranial aneurysm (UIA) may face an increased risk of UIA rupture. This study investigated the rupture risk of UIA in patients with VM and proposed a plausible explanation for the associations between VM, migraine, and UIA distribution, particularly concerning interactions within vascular and nociceptive conduction pathways.

**Methods:**

A cross‐sectional case‐control study involving 148 subjects diagnosed with UIA was conducted, who were categorized into three groups: the VM, the migraine, and the control groups. The distribution of parent arteries and the morphological parameters of the UIA, such as diameter, size, depth, neck width, mean parent arterial diameter, size ratio, non‐spherical index (NSI), and parameters specific to bifurcation UIA, were extracted from the original imaging data and 3D‐Slicer software for intergroup comparison.

**Results:**

UIA was predominantly located in the internal carotid arteries (ICA) at C4 (12.3%), C5 (14.0%), and C7 (35.1%) in the VM group. The migraine group exhibited UIA primarily in the ICA at C6 (42.2%) and the vertebrobasilar artery (10.9%). In the control group, UIA was concentrated in the middle cerebral artery (22.6%). These distributions differed significantly (*p_1_* = 0.002, *p_3_* = 0.017). Furthermore, a statistically significant difference was observed in the NSI between the VM and migraine groups (*p* = 0.044).

**Conclusions:**

We observed significant differences in the distribution of UIA between the VM and migraine groups compared to the control group. Furthermore, intergroup comparisons of morphological parameters indicated that both VM and migraine patients have a higher risk of aneurysm rupture. We propose a plausible hypothesis regarding the relationship between VM, migraine, and UIA distribution. Future research should involve more precise hemodynamic analyses, long‐term patient follow‐up, and potential animal studies.

## Graphical abstract

We observed significant differences in the distribution of unruptured intracranial aneurysm (UIA) between the vestibular migraine (VM) and migraine groups compared to the control group. Furthermore, intergroup comparisons of morphological parameters indicated that both VM (non‐spherical index) and migraine (age) patients have a higher risk of aneurysm rupture.

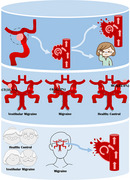

## 1 Introduction

**FIGURE 1:**
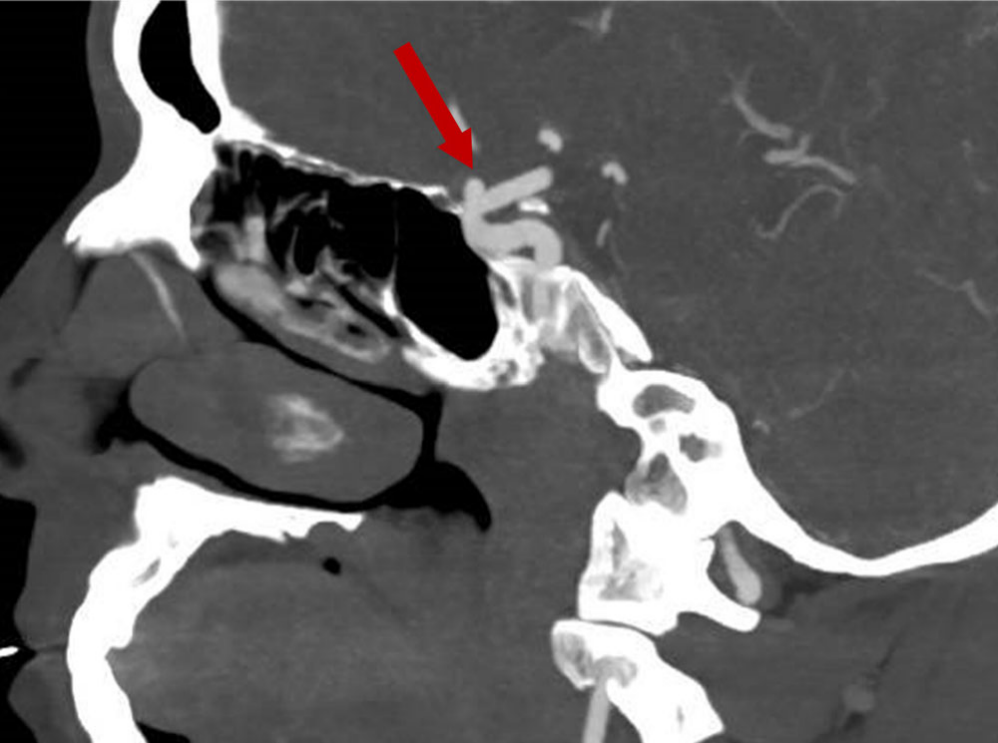
Ophthalmic segment ICA aneurysm (indicated by red arrow).

Vestibular migraine (VM), previously referred to as “migraine‐associated vertigo/dizziness,” is a vestibular episodic disorder that can be triggered by various factors, including menstruation, lack of sleep, and certain foods. It is more prevalent in females and can occur at any age. Intracranial aneurysm (IA) is defined as an abnormal dilation of the intracranial arteries resulting from a gradual, localized, and permanent expansion due to the combined effects of congenital developmental abnormalities and damage caused by various acquired factors, including hemodynamic stress (Figure 1). Headache and vertigo are among the most common symptoms reported by patients with unruptured intracranial aneurysms (UIA), with prevalence rates of 46% and 21%, respectively. Another study reported corresponding rates were 46.1% for headache and 80.9% for vertigo (Hackett et al. 2023, Gkasdaris et al. 2025). The majority of UIAs are incidentally identified through magnetic resonance angiography (MRA) and computed tomography angiography (CTA) during presentation with chronic headache and dizziness. (Gabriel et al. 2010) Prior research has established a correlation between VM, migraine, and an increased prevalence of vascular abnormalities, including IA (Witvoet et al. 2017), Patent Foramen Ovale (PFO) (Hou et al. 2018, Wei et al. 2021, Liu et al. 2020, Zhang et al. 2021, Liu et al. 2022), Spontaneous Coronary Artery Dissection (SCAD) (Kok et al. 2018), Fibromuscular Dysplasia (FMD), and others. Several studies have confirmed or hypothesized that the higher prevalence of IA in migraine patients may be attributed to endothelial dysfunction and prolonged exposure to vasoactive peptides, which compromise vascular wall integrity (Noseda and Pathophysiology 2013). Furthermore, the prevalence of aneurysmal subarachnoid hemorrhage (aSAH) is significantly elevated in UIA patients with migraine, owing to a higher prevalence of aneurysms, an increased risk of rupture, or both. (Witvoet et al. 2017). In clinical practice at the Vertigo Center, we have observed that UIA in patients with VM and migraine exhibits distinct distributions of parent arteries and IA morphology compared to those in patients without such a history. Furthermore, these patients experience more severe symptoms and more frequent episodes than those without the aforementioned history. VM shares similarities with migraine in its etiology and is a variant characterized by vestibular manifestations. Consequently, this study aims to examine the risk of UIA rupture in patients with VM by analyzing morphological parameters and propose a plausible explanation for the association between VM and UIA from the perspectives of vascular and nociceptive conduction pathways, ultimately establishing a clinical research framework for further investigation.

## 2 Methods

### 2.1 Patient Selection

A retrospective analysis was performed on patients who attended the Second Affiliated Hospital of Zhengzhou University between January 2018 and June 2024.

#### 2.1.1 Inclusion Criteria for the VM and Migraine Groups

Patients older than 18 years of age; patients meeting the diagnostic criteria for VM (2022) and migraine (2018) as outlined in the Consensus document of the Bárány Society and the International Headache Society (IHS) (Lempert et al. 2022) and the International Headache Association (ICHD‐III) (Headache Classification Committee of the International Headache Society (IHS), the International Classification of Headache Disorders 2018); a first diagnosis of IA confirmed by at least one of the following imaging modalities: CTA, MRA, or DSA.

#### 2.1.2 Inclusion Criteria for the Control Group

Patients older than 18 years of age; no history of migraine or VM; a first diagnosis of IA confirmed by at least one of the following imaging modalities: CTA, MRA, or DSA.

#### 2.1.3 Exclusion Criteria

A history of subarachnoid hemorrhage; secondary intracranial aneurysm resulting from infection, trauma, or other causes; comorbidities involving other cerebrovascular diseases, including Moyamoya disease, intracranial arteriovenous malformations, and arteriovenous fistulas; evidence of vascular infundibular changes or other suspicious vascular morphologies, such as arterial cones or arterial ampullas; a familial history of IA in two or more family members; other potential causes of vertigo such as Benign paroxysmal positional vertigo (excluded by negative Dix–Hallpike test, Ménière's disease (ruled out by audiometry and Gadolinium‐Enhanced MRI), Vestibular neuritis excluded by video head impulse testing, posterior circulation stroke (eliminated by neuroimaging) as well as headache, including Cluster headache or Tension‐type headache (diagnosed by clinical criteria).

### 2.2 Data Collection

The baseline clinical characteristics of the subjects, including age, sex, comorbidities, and smoking history, were recorded. Additionally, data on the UIA distribution, laterality (left or right), morphology (saccular or irregular, with lobulated structures or daughter blebs), distribution of parent arteries, and morphological parameters (diameter, size, depth, neck width, mean parent arterial diameter, area of the plane of measurement, and volume of the UIA) were documented. Furthermore, size ratio, the non‐spherical index (NSI), incidence angle, and diameter ratio in bifurcation UIA, as well as the bifurcation angle, were measured.

### 2.3 Classification and Definitions of Unruptured Intracranial Aneurysms

#### 2.3.1 Primary Aneurysms

1. Saccular (berry) aneurysms are focal outpouchings of the arterial wall characterized by a distinct neck and dome, most frequently found at arterial bifurcations.
2. Fusiform aneurysms involve circumferential dilation of the arterial wall without a defined neck, often associated with atherosclerosis or vasculitis.
3. Dissecting aneurysms are defined by vascular intimal tears resulting in intramural hematoma formation, demonstrating the pathognomonic “double lumen” sign on high‐resolution magnetic resonance imaging (HR‐MRI).

#### 2.3.2 Secondary Intracranial Aneurysms

1. Traumatic intracranial aneurysms most commonly involve the middle meningeal artery (MMA) or cavernous segment (C4) of the internal carotid artery (ICA). These pseudo aneurysms are caused by arterial wall disruption, with delayed contrast extravasation on angiography.
2. Infectious aneurysms result from septic embolization or direct microbial invasion of the vessel wall, leading to inflammatory destruction and irregular aneurysmal morphology.
3. Pseudo aneurysms result from partial disruption of the arterial wall, with blood extravasation contained by surrounding tissues. They have a true endothelial lining and are predominantly secondary to trauma or infection.

### 2.4 Definitions of the Morphological Parameters

**FIGURE 2:**
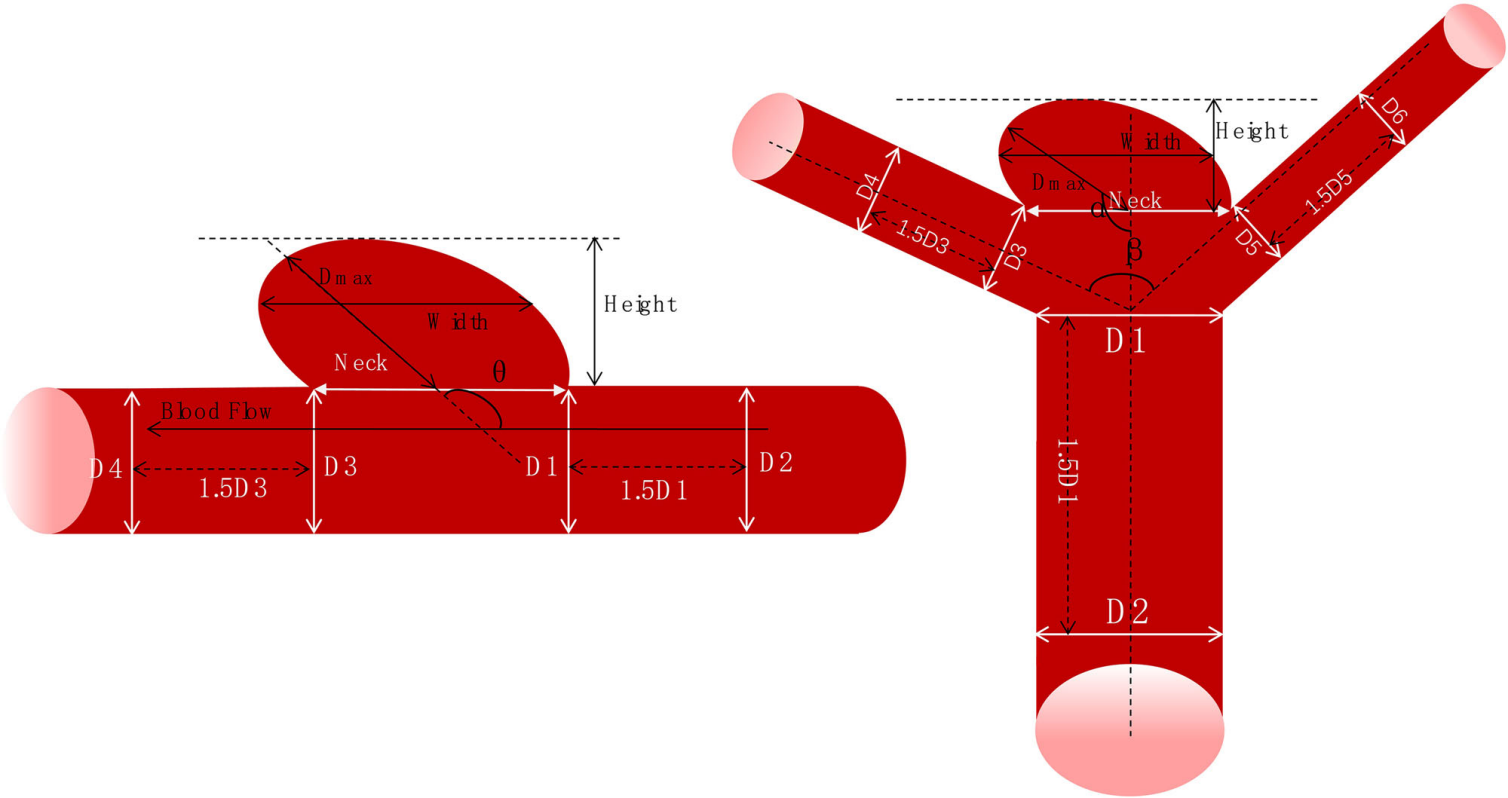
Schematic diagram defining morphological parameters of intracranial sidewall and bifurcation aneurysms.

The detailed definitions of the morphological parameters are provided below and depicted in Figure 2.

1. Diameter (*D _max_*): The maximum distance from the midpoint of the neck plane to the aneurysm apex;
2. Size/Height: The greatest vertical height of the aneurysm to the neck plane, defined as the transverse section that separating the aneurysm from the parent vessel;
3. Width: The maximum horizontal distance parallel to the neck plane;
4. Neck width (Neck): The maximum diameter at the junction between the aneurysm and the parent artery;
5. Mean parent arterial diameter (*D _ave_*): the average diameter at the aneurysm‐parent artery junction and 1.5 times the distance distal to this point.

For side‐wall aneurysm, $Dave=\frac{D1+D2+D3+D4}{4}$;

For bifurcation aneurysm, $Dave=\frac{D1+D2+D3+D4+D5+D6}{6}$;

6. Size Ratio (SR): The ratio of the maximum vertical height to the mean parent artery diameter, $SR=\frac{Height}{D\;ave}$
7. NSI: Quantifies the deviation from a perfect sphere.
$$NSI=1-{\left(18\pi \right)}^{\frac{1}{3}}\frac{V^{\frac{2}{3}}}{S},S=Height\times Width$$
  where V is the aneurysm volume extracted and calculated from 3D‐Slicer software and S is its planar area.

8. Aspect Ratio (AR): $\mathrm{AR}\;=\frac{\mathrm{Dmax}}{\mathrm{Neck}\;\mathrm{width}}$;
9. Incidence Angle:

For side‐wall aneurysm, the angle θ between the aneurysm centerline and the neck plane;

For bifurcation aneurysm, the angle α between the pre‐bifurcation artery centerline and the aneurysm centerline.

10. Diameter Ratio (in bifurcation UIA): $\frac{D1+D2}{D3+D4+D5+D6}$;
11. Bifurcation angle: The angle β between the centerline extensions of the two branch arteries.

Following the seven‐segment segmentation method of the internal carotid artery proposed by Bouthillier et al. in 1996, the parent arteries were divided into ICA segments C1‐C7, the anterior cerebral artery (ACA), the anterior communicating artery (AcoA), the middle cerebral artery (MCA), the posterior communicating artery (PcoA), the posterior cerebral artery (PCA), and the vertebral basilar artery (VBA).

### 2.5 Imaging and Measurement Protocols

#### 2.5.1 Imaging Equipment

The MRI equipment model utilized in this study was the SIEMENS MAGNETOM Lumina 3.0T with the following parameters: 3D TOF MRA sequence, Repetition Time (TR) = 19.4 ms, Time to Echo (TE) = 3.69 ms, Flip Angle = 20°, Slice Thickness = 0.5 mm. The CT equipment employed was the GE HealthCare 64‐row Revolution EVO with standardized protocols: tube voltage = 120 kV, tube current = 450 mA, slice thickness = 1.25 mm, and iodinated contrast agent (Iodixanol Injection, 32 mgI/100 mL) administered at 4.7 mL/s. The DSA equipment model was the PHILIPS Azurion 3 M15 with acquisition parameters of 2 frames per second and iodinated contrast injection (Iomeprol, Italy. S.p.A. 30 mgI/100 mL) rates tailored to vascular anatomy (e.g., 5 mL/s for ICA, 4 mL/s for VBA).

#### 2.5.2 Image Analysis

Each participant underwent either CTA, MRA, or DSA based on clinical indications, but not both. The selection of imaging modality was guided by individualized patient factors such as renal function, presence of metallic implants, and clinical judgment. The UIA images acquired from the MRA and CTA examinations were analyzed for length and angle measurements using the Picture Archiving and Communication System (PACS) measurement tools. Three‐dimensional model reconstructions and IA volume measurements were conducted using the 3D‐Slicer software, which reconstructs two‐dimensional DICOM images from CT or MRI into 3D models and enables multimodal image fusion. The measurement procedure was supervised by two experienced imaging technicians and carried out independently by a single member of the study team. Each parameter was measured three times, with the average value taken as the final data.

#### 2.5.3 3D‐Slicer Procedure

**FIGURE 3:**
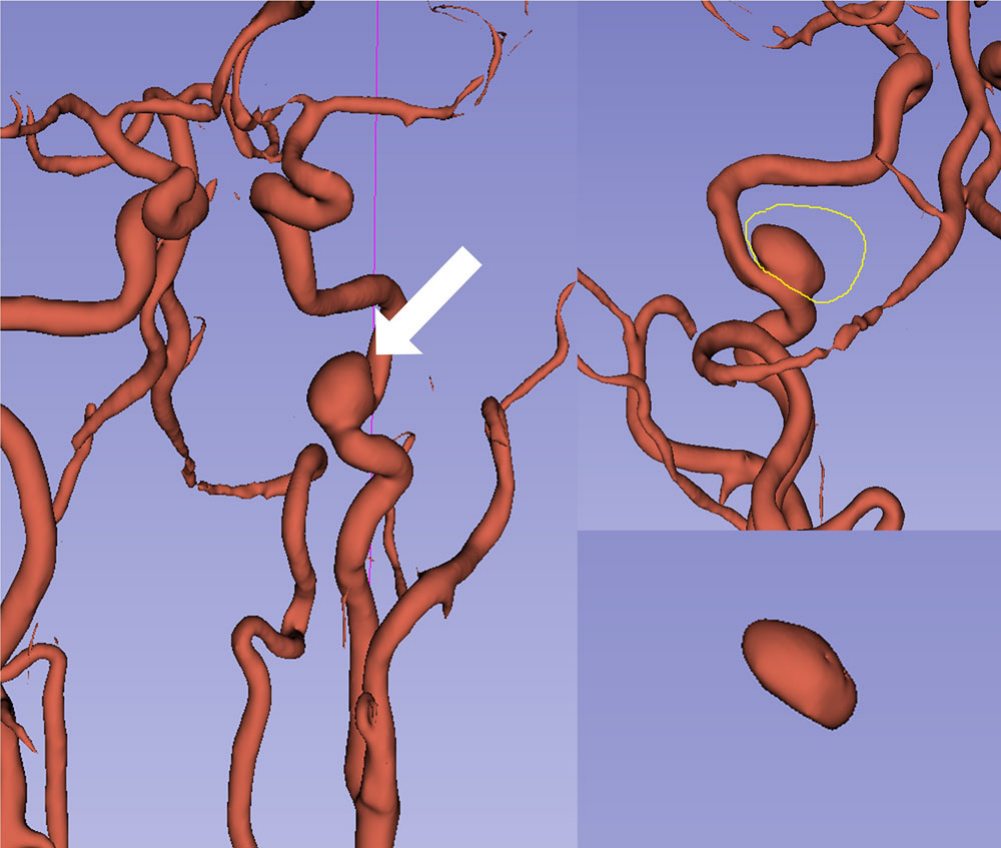
Volumetric measurement of C3 segment ICA aneurysm using 3D‐Slicer software.

Studies have demonstrated that 3D‐Slicer exhibits high sensitivity and specificity in diagnosing IA, comparable to DSA, which is considered the gold standard for diagnosis, with no statistically significant difference in assessing aneurysm size. (Jiang et al. 2019, Pei et al. 2019, Chalouhi et al. 2012) Therefore, the results obtained from reconstructing the cerebral arteries and deriving the morphological parameters via 3D‐Slicer are both accurate and reliable. The specific procedure for the analysis was as follows: 1) import the raw data from the cranial CTA and MRA into 3D Slicer software (Windows 11, version 4.10.2) in DICOM format; 2) utilize the “Segment Editor” module, select “Add” and “Threshold,” and establish a suitable range of density values to cover the intracranial arteries; 3) switch on “Use for masking” and apply the “Paint” function to delineate the arteries; 4) select “Show 3D” to generate a complete and clear 3D model of the intracranial arteries and the UIA; 5) accurately segment the IA using the “Scissors” tool after precise positioning. Finally, select “Data,” then “Export visible segment to models,” and read the volume data in the “Models” module, as illustrated in Figure 3.

#### 2.5.4 DSA Measurements

**FIGURE 4:**
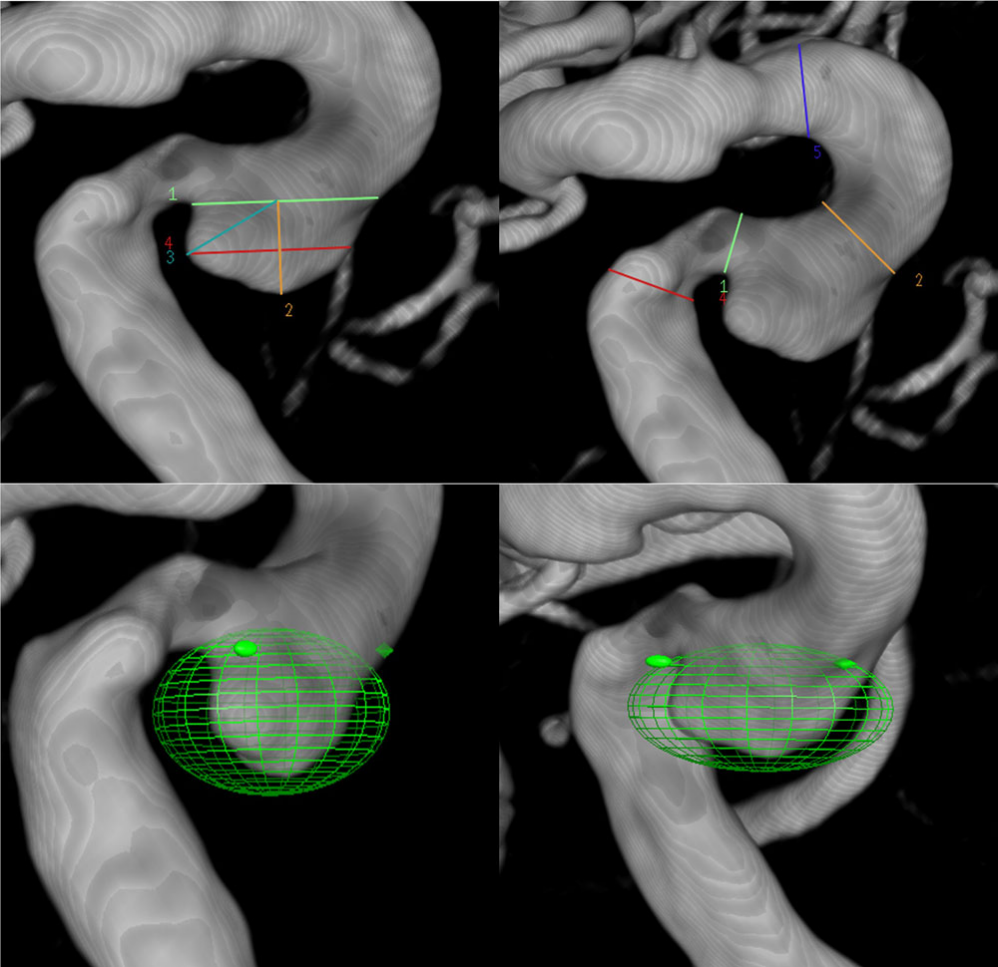
Diagram of UIA morphological parameters and volume measurement in DSA post‐processing workstation.

The DSA parameters were measured using the PHILIPS Azurion system post‐processing workstation. Specifically, the “Quick Measurement,” “Snapshot,” and “Volume Measurement” features were employed for length, angle, and volume assessments, respectively. Volume measurement (Figure 4) was conducted within an adjustable elliptical area, ensuring that the UIA under examination was entirely encompassed within the measurement area without including the parent artery.

### 2.6 Statistical Techniques

All data were statistically analyzed using SPSS version 25.0 (IBM, Armonk, NY, USA). For categorical data, presented as absolute values and percentages, the chi‐square test and Fisher's exact probability method were utilized for between‐group comparisons. For continuous data, normality was assessed using the Shapiro‐Wilk test. Data that followed a normal distribution were expressed as the mean ± standard deviation (SD) and compared between groups using analysis of variance (ANOVA); multiple comparisons were conducted using the least significant difference (LSD) method. Data that did not conform to a normal distribution were expressed as median (quartiles) [M‐(Q25, Q75)] and compared between groups using the Kruskal–Wallis test. Statistical significance was accepted at *p* < 0.05.

### 2.7 Ethical Approval

This study was approved by the Ethics Committee of the Second Affiliated Hospital of Zhengzhou University. Written informed consent was obtained from each subject.

## 3 Results

### 3.1 Baseline Data Analysis

A total of 148 subjects (mean age: 55.65 years; 70.9% female) were enrolled. The migraine group had a significantly younger age at the first detection of UIA compared to the VM and control groups (*p_1_* = 0.015, *p_2_* < 0.001); following age stratification, 80.71% of the migraine group had their UIA detected before the age of 60, significantly higher than the VM (54.54%) and control groups (51.07%) (*p_1_* = 0.014, *p_2_* = 0.001). Furthermore, the prevalence of diabetes mellitus was significantly higher in the VM (18.18%) and control groups (19.15%) than in the migraine group (5.26%). The incidence of hyperlipidemia was also significantly higher in the VM group (63.64%) compared to the migraine (35.09%) and control groups (31.91%). Gender and a history of hypertension or smoking did not differ significantly among the groups, as presented in Table 1.

**TABLE 1:** Results of univariate analysis of clinical baseline data for the three groups of subjects.

Items (cases/%)	VM (*n* = 44)	Migraine (*n* = 57)	Control (*n* = 47)	*p*_1_	*p*_2_	*p*_3_
Gender						
Male	10 (22.73)	18 (31.58)	15 (31.91)	0.375	1.000	0.357
Female	34 (77.27)	39 (68.42)	32 (68.09)			
Age	58.0 (49.0,66.0)	52.0 (43.0,58.5)	61.0 (55.0,68.0)	0.015^*^	< 0.001^*^	0.309^*^
Age group						
≤ 50	15 (34.09)	24 (42.11)	6 (12.77)	0.014	0.001	0.071
51‐60	9 (20.45)	22 (38.60)	18 (38.30)			
61‐70	16 (36.36)	6 (10.53)	19 (40.43)			
> 70	4 (9.09)	5 (8.77)	4(8.51)			
Hypertension	14 (31.82)	12 (21.05)	16(34.04)	0.220	0.137	0.822
Diabetes	8 (18.18)	3 (5.26)	9(19.15)	0.039	0.027	0.906
Hyperlipidemia	28 (63.64)	20 (35.09)	15(31.91)	0.004	0.733	0.002
Smoking history	8 (18.18)	9 (15.79)	6(12.77)	0.750	0.662	0.474

*p*_1_, *p*_2,_ and *p*_3_ denote the *p*‐values for comparisons between the VM and migraine groups, the migraine and control groups, and the VM and control groups, respectively. *Adjusted significance.

### 3.2 Distribution Analysis

The distribution of the parent arteries associated with all 183 cases of UIA is presented in Table 2. In the VM group, UIA developed more significantly in the ICA, specifically in the cavernous segment (C4, 7/57, 12.3%), clinoid segment (C5, 8/57, 14.0%), and communicating segment (C7, 20/57, 35.1%). In the migraine group, UIA were significantly more common in the ICA at the ophthalmic segment (C6, 27/64, 42.2%) and in the VBA (7/64, 10.9%). In the control group, UIA were more prevalent in the MCA (14/62, 22.6%). A statistically significant difference was observed in the distribution of UIA between the VM group and both the migraine and control groups (*p_1_* = 0.002, *p_3_* = 0.017). After excluding sites with similar proportions of UIA between the migraine and control groups, significant differences remained in the distribution of UIA in the C6 of ICA and the MCA between these two groups.

**TABLE 2:** Distribution of UIA parent arteries among the three groups.

Items (cases/%)	VM (*n* = 44)	Migraine (*n* = 57)	Control (*n* = 47)	*p*_1_	*p*_2_	*p*_3_
ACA	2 (3.51)	5 (7.81)	5 (8.06)			
AcoA	2 (3.51)	2 (3.13)	2 (3.23)			
C2	0 (0.0)	0 (0.0)	1 (1.61)			
C3	0 (0.0)	1 (1.56)	1 (1.61)			
C4	7 (12.28)	4 (6.25)	4 (6.45)			
C5	8 (14.04)	2 (3.13)	5 (8.06)			
C6	11 (19.30)	27 (42.19)	14 (22.58)			
C7	20 (35.09)	10 (15.63)	9 (14.52)			
MCA	4 (7.02)	6 (9.38)	14 (22.58)			
PCA	0 (0.0)	0 (0.0)	1 (1.61)			
PcoA	2 (3.51)	0 (0.0)	0 (0.0)			
VBA	1 (1.75)	7 (10.94)	6 (9.68)			
Total	57	64	62	0.002	0.327	0.017

*p_1_*, *p_2,_* and *p_3_* denote the *p*‐values for comparisons between the VM and migraine groups, the migraine and control groups, and the VM and control groups, respectively.

### 3.3 Morphological Parameters Analysis

Out of the 183 UIAs, 8 (1 in the VM group, 5 in the migraine group, and 2 in the control group) were excluded from the statistical analyses of morphological parameters due to damaged or missing imaging data. The morphological characteristics of UIA parameters are presented in Table 3. Notably, the differences in UIA image acquisition methods among the groups were statistically significant (*p* = 0.003). The most common two‐dimensional DICOM file acquisition methods in the VM and migraine groups were CTA and MRA, whereas DSA was more prevalent in the control group.

**TABLE 3:** Morphological parameters of UIAs in three groups of participants.

Items	VM (n = 44)	Migraine (n = 57)	Control(n = 47)	*p*_1_	*p*_2_	*p*_3_
Examination methods (case/%)*						
CTA	18 (40.91)	28 (49.12)	9 (19.15)	0.003		
MRA	18 (40.91)	13 (22.81)	15 (31.91)			
DSA	8 (18.18)	16 (28.07)	23 (48.94)			
Side (case/%)						
Right	34 (62.96)	32 (51.61)	27 (46.55)	0.218	0.579	0.081
Left	20 (37.04)	30 (48.39)	31 (53.45)			
Morphology (case/%)						
regular	43 (76.79)	46 (77.97)	46 (74.19)	0.880	0.627	0.744
Irregular	13 (23.21)	13 (22.03)	16 (25.81)			
Orientation (case/%)						
Front	19 (33.93)	25 (43.10)	28 (46.67)	0.314	0.697	0.163
Back	37 (66.07)	33 (56.90)	32 (53.33)			
Inside	30 (54.55)	37 (64.91)	38 (65.51)	0.263	0.946	0.234
Outside	25 (45.45)	20 (35.09)	20 (34.48)			
Upper	14 (25.00)	19 (32.76)	27 (45.00)	0.361	0.173	0.024
Lower	42 (75.00)	39 (67.24)	33 (55.00)			
Incidence Angle (°)	76.79 ± 31.56°	82.27 ± 31.15°	80.31 ± 30.56°	0.370	0.747	0.566
>90°(case/%)	15 (29.41)	16 (30.19)	16 (30.76)	0.931	0.948	0.881
Diameter (mm)	2.40 (1.90, 3.18)	2.80 (2.12, 3.40)	2.89 (2.00, 3.91)	0.133		
Small (case/%)	49 (87.50)	54 (91.53)	51 (85.00)	0.518		
Medium (case/%)	6 (10.71)	5 (8.47)	9 (15.00)			
Large (case/%)	1 (1.79)	0 (0.0)	0 (0.0)			
Huge (case/%)	0 (0.0)	0 (0.0)	0 (0.0)			
Size Ratio (case/%)						
>2	2 (3.92)	2 (3.77)	3 (5.77)	0.898		
≤2	49 (96.08)	51 (96.23)	49 (94.23)			
Aspect Ratio	0.56 (0.46, 0.82)	0.57 (0.30, 0.74)	0.65 (0.37, 0.89)	0.217		
Volume(mm3)	17.94 (11.11,36.51)	20.03 (9.71,48.30)	27.10 (13.21,71.45)	0.101		
Non‐spherical index	−24.66 (−33.02, −15.68)	−18.08 (−28.24, −11.93)	−22.84 (−27.79, −17.88)	0.044	0.369	1.000
bifurcation UIA						
Diameter before bifurcation	2.89 ± 0.84	2.84 ± 0.79	3.05 ± 1.23	0.938	0.714	0.791
Diameter after bifurcation	2.57 ± 0.54	2.02 ± 0.59	2.27 ± 0.84	0.215	0.515	0.469
Diameter ratio	1.19 (0.98, 1.23)	1.32 (1.15, 1.80)	1.27 (1.14, 1.37)	0.262**		
Incidence angle	149.14 ± 25.00°	144.18 ± 13.56°	145.35 ± 29.30°	0.740	0.930	0.788
Bifurcation angle	164.80 ± 36.69°	152.47 ± 62.39°	151.35 ± 40.00°	0.674	0.966	0.626
Non‐spherical index	−26.84 (−85.51, −13.15)	−20.57 (−29.17, −8.77)	−23.42 (−30.92, −20.93)	0.522		

*p_1_*, *p_2,_* and *p_3_* denote the *p*‐values for comparisons between the VM and migraine groups, the migraine and control groups, and the VM and control groups, respectively. * *Note*: Single imaging modality per participant (CTA/MRA/DSA). **Adjusted significance.

After excluding non‐lateral UIA, no statistically significant differences were observed in the lateralization of UIA among the groups. Additionally, there were no statistically significant differences in the percentage of morphologically irregular UIA, nor were there differences in orientation, incidence angle, diameter, size ratio, volume, and aspect ratio of UIA. In the bifurcation aneurysms, no significant differences in morphological parameters were observed between the groups. Notably, the VM group had a significantly higher proportion of downward‐oriented aneurysms (42/56, 75%) than the control group. Furthermore, the NSI in the VM group significantly differed from that in the migraine group.

### 3.4 Potential Influence of Imaging Modalities

In accordance with the reviewers' suggestions, we conducted additional analyses to evaluate the potential influence of imaging modalities on study outcomes. Participants were stratified into three groups based on imaging techniques: MRA, CTA, and DSA groups, to examine differences in demographic characteristics (Table 4), parent artery distribution (Table 5), and key morphological parameters of UIA (Table 6).

**TABLE 4:** Clinical baseline data among different imaging modalities.

Items (cases/%)	MRA (n = 46)	CTA (n = 55)	DSA (n = 47)	*p*_1_	*p*_2_	*p*_3_
**Gender**						
**Male**	11 (23.91)	17 (30.91)	15 (31.91)		0.648	
**Female**	35 (76.09)	38 (69.09)	32 (68.09)			
**Age**	55.43 ± 16.08	54.87 ± 11.20	54.98 ± 12.38	0.837	0.964	0.878
**Hypertension**	15 (32.61)	17 (30.91)	10 (21.28)		0.418	
**Diabetes**	8 (17.39)	7 (12.73)	5 (10.64)		0.621	
**Hyperlipidemia**	20 (43.48)	23 (41.82)	20 (42.55)		0.986	
**Smoking history**	6 (13.04)	7 (12.73)	10 (21.28)		0.421	

*p_1_*, *p_2,_* and *p_3_* denote the *p*‐values for comparisons between the MRA and CTA groups, the CTA and DSA groups, and the MRA and DSA groups, respectively.

**TABLE 5:** Distribution of UIA parent arteries among different imaging modalities.

Items (cases/%)	MRA (n = 55)	CTA (n = 68)	DSA (n = 60)	*p*_1_	*p*_2_	*p*_3_
**ACA**	4 (7.27)	3 (4.41)	5 (8.33)			
**AcoA**	3 (5.45)	1 (1.47)	2 (3.33)			
**C2**	0 (0.0)	0 (0.0)	1 (1.67)			
**C3**	1 (1.82)	0 (0.0)	1 (1.67)			
**C4**	7 (12.7)	5 (7.35)	3 (5.00)			
**C5**	7 (12.7)	6 (8.82)	2 (3.33)		0.490	
**C6**	11 (20.00)	21 (30.88)	21 (35.00)			
**C7**	12 (21.82)	14 (20.59)	13 (21.67)			
**MCA**	4 (7.27)	13 (19.12)	7 (11.67)			
**PCA**	0 (0.0)	0 (0.0)	1 (1.67)			
**PcoA**	0 (0.0)	1 (1.47)	0 (0.0)			
**VBA**	6 (10.91)	4 (5.88)	4 (6.67)			

*p_1_*, *p_2_* and *p,_3_* denote the *p*‐values for comparisons between the MRA and CTA groups, the CTA and DSA groups, and the MRA and DSA groups, respectively.

**TABLE 6:** key UIA morphological parameters among different imaging modalities.

Items	MRA (n = 55)	CTA (n = 68)	DSA (n = 60)	*p*_1_	*p*_2_	*p*_3_
Diameter (mm)	2.25 (1.90, 2.88)	2.73 (2.19, 3.40)	3.32 (1.91, 4.55)	0.004	0.199	0.005
Incidence Angle (°)	76.80 (60.30, 109.70)	77.35 (57.73, 107.25)	89.70 (68.75, 113.63)	0.786	0.234	0.139
Volume (mm^3^)	18.83 (8.88,3 0.82)	20.46 (11.14, 47.61)	27.00 (11.00, 93.00)	0.393	0.135	0.032
Non‐spherical index	−23.67 (‐30.20, ‐17.38)	−17.57 (‐26.80, ‐11.81)	−22.94 (‐32.50, ‐18.10)	0.051	0.007	0.649

*p_1_*, *p_2,_* and *p_3_* denote the *p*‐values for comparisons between the MRA and CTA groups, the CTA and DSA groups, and the MRA and DSA groups, respectively.

The results revealed no significant differences among the three groups in terms of age, gender, comorbidities, or smoking history, nor in the distribution of parent arteries. However, significant intergroup differences were observed in aneurysm diameter (MRA vs. CTA, *p* = 0.004; MRA vs. DSA, *p* = 0.005). Additionally, the MRA group exhibited a significantly smaller aneurysm volume compared to the DSA group (*p* = 0.032), and the NSI differed significantly between the CTA and DSA groups (*p* = 0.007).

## 4 Discussion

In this study, we analyzed the baseline clinical data and morphological parameters of UIA patients combined with VM. We identified significant differences in the distribution of IA among patients with VM, migraine, and those in the control group. These locations may represent potential sites of inherent developmental weakness or heightened susceptibility to pathological processes. Additionally, by analyzing morphological parameters, we found a significantly elevated risk of rupture associated with the UIA in the VM and migraine groups. Our findings may provide valuable insights for clinical screening and the development of diagnostic and therapeutic strategies. Previous studies (Witvoet et al. 2017) have confirmed that the incidence of IA in migraine patients is higher than in the general population. Although there are no reports specifically addressing VM, we deduced that patients with VM exhibit a high incidence of UIA. This study did not further investigate the incidence of IA.

The VM, migraine, and control groups predominantly comprised female, middle‐aged, and elderly patients, consistent with existing epidemiological data on VM and migraine, as well as previous studies on IA. The age at which UIA was detected in migraine and VM patients was younger than in the control group. Recent investigations into small intracranial aneurysms have identified age under 50 years as an independent risk factor for rupture (Sonobe et al. 2010, Güresir et al. 2013). Our study found that the migraine group was significantly younger, with the highest proportion of patients aged under 50 years. Consequently, we concluded that the rupture risk of UIA is higher in the migraine population, aligning with the findings of Witvoet et al. (2017). Hypertension and smoking have long been recognized as key factors related to the occurrence and progression of IA, contributing to an increased risk of IA formation and rupture (Tutino et al. 2015, Jeon et al. 2014). However, our study showed no statistically significant difference in the history of hypertension and smoking among the three groups. Bir et al. (2015) have demonstrated a significant association between diabetes mellitus and IA growth and rupture. Conversely, Lindgren et al. (2013) and Vlak et al. (2013) found no increase in the risk of IA rupture associated with diabetes. Additionally, hyperlipidemia has been shown to affect the vessel walls, and Bir et al. (2015) indicated a correlation between hyperlipidemia and IA formation and rupture. In contrast, Inagawa (2010) concluded that hyperlipidemia significantly reduces the risk of IA rupture. Although there is currently no consensus on whether diabetes and hyperlipidemia promote the formation, progression, and rupture of IA, morphological parameter analyses revealed that NSI in the VM group was significantly greater than that in the migraine group (*p* = 0.044), which illustrates that UIAs in the VM group displayed a tendency to deviate from a perfect spherical shape and exhibited a higher risk of rupture compared to those in the migraine group. Therefore, in the absence of significant differences in hypertension and smoking history, diabetes mellitus and hyperlipidemia are potential predisposing factors for the development of IA and the increased risk of rupture in the VM group. Furthermore, deviation from a spherical geometry may intensify hemodynamic disturbances such as vortex formation during systolic deceleration, which disrupts laminar flow in the parent artery (Gkasdaris et al. 2021). These alterations could potentially impair cerebral autoregulation and exacerbate vestibular symptoms. (Gkasdaris et al. 2025)

The injection of neuronal tracers into mouse models has confirmed that the vestibular nucleus receives afferent signals from the trigeminal nerve and is involved in the migraine pathway (Buisseret‐Delmas et al. 1999, Porter and Balaban 1997, Diagne et al. 2006, Menétrey and Basbaum 1987, Balaban and Beryozkin 1994, Yates et al. 1994). Studies have revealed that electrical stimulation of high pain in the forehead, aimed at activating the trigeminal nerve, induces spontaneous nystagmus in patients with VM but not in the control group. Furthermore, research has shown that the severing of the trigeminal branches of the intracranial vasculature significantly reduces the frequency of migraine attacks and alleviates their severity (Schnell et al. 2014). It is hypothesized that the formation of IA in specific areas of VM or migraine patients leads to alterations in hemodynamics. This change stimulates receptors in the vascular wall, activates the ophthalmic branch of the trigeminal nerve, and concurrently impacts the vestibular nuclei. Additionally, it disrupts the functioning of the nuclei in the nerve region supplied by blood and the associated fiber bundles. Ultimately, this results in an exacerbation of vertigo and headache perception in the thalamus and cerebral cortex. In clinical practice, site‐specific UIA may be identified as a potential etiological factor in refractory VM and migraine.

In light of the pathomechanism of VM proposed by Furman et al. (2005, 2013), it can be inferred that, in the absence of obvious vestibular afferents, UIA in arteries supplying blood to various segments of the vestibular conductive pathway do not exacerbate VM symptoms or the frequency of seizures. Conversely, UIA in arteries supplying blood to segments of the cephalofacial nociceptive conductive pathway may lead to peripheral trigeminal excitability and dysfunction within these pathways, ultimately worsening VM symptoms and increasing seizure frequency. Whether the hemodynamic changes caused by UIA will affect the function of the nerve nuclei and fiber tracts should be assessed in relation to the diameter of the vessels and the distance between the UIA region and the blood‐supplying nuclei. For instance, the trigeminal ganglion receives blood from the artery of the inferior cavernous sinus, which originates from the C4 segment of the internal carotid artery. In 95% of cases, the ophthalmic branch of the trigeminal nerve is supplied by the inferior lateral cavernous sinus artery. Furthermore, central thalamus radiation is supplied by perforators of the internal carotid artery originating from the C5 segment in the posterior limb of the internal capsule, as well as by choroidal arteries from the C7 segment of the internal carotid artery. Due to the considerable traveling distance and diminutive diameter of these arterial branches, UIAs in these locations, triggering hemodynamic changes, can disrupt conductive pathways and activate trigeminal and vestibular nerve nuclei due to stimulation of the arteries as intracranial nociceptive sensitive tissues. This can lead to the exacerbation of VM symptoms and an increase in seizure frequency.

The C6 segment of the internal carotid artery supplies blood to the eye. The posterior inferior cerebellar artery (PICA), the largest branch of the vertebrobasilar system, is responsible for supplying blood to the vestibular nucleus and the nucleus of the spinal trigeminal nucleus. Given the ample diameter of PICA, UIA are unlikely to significantly affect the functions of these nuclei. Furthermore, the observed differences between the migraine group and the other groups may be due to the impact of a single participant with multiple vertebral artery UIAs. The worsening of migraine symptoms and increased attack frequency associated with UIAs at the aforementioned sites could stem from the peripheral nerve activation of the trigeminal nerve. Additionally, the cortical branches of the middle cerebral artery, which have considerable diameters, supply the vestibular cortex (insular cortex) and the lower third of the postcentral gyrus. However, the UIAs identified in this study were located in the M1 and M2 segments and the bifurcation of M1, all of which are substantially distant from the insular cortex and the postcentral gyrus, thus unlikely to influence their function.

Further refinement of hemodynamic analyses, prolonged follow‐up of patients with VM and migraine, and additional animal experimentation are necessary to substantiate this hypothesis.

Previous studies have consistently demonstrated that intracranial aneurysms occur significantly more often in the anterior circulation than in the posterior circulation (Vlak et al. 2011). Population‐based studies report the following distribution: MCA (35%), ACA (18%), ICA (42%), PCoA (10%), and VBA (5%). Notably, aneurysm location is strongly associated with rupture risk (Ogilvy 2025). Based on six prospective cohort studies using MCA as reference (Hazard Ratio, HR = 1.0), ICA aneurysms show the lowest rupture risk (HR = 0.5), while ACA (HR = 1.8), ACoA (HR = 2.1), and PCoA (HR = 1.6) aneurysms demonstrate significantly higher rupture propensity.

We acknowledge that the proportions of AcoA (3.13–3.51%) and MCA (7.02–22.58%) aneurysms in our cohort were lower than those reported in some population‐based studies. This discrepancy may be attributed to the following factors: 1) Insufficient sample size and selection bias. 2) The inherent lower rupture risk of ICA aneurysms (HR 0.5) increases their representation in UIA screening cohorts. Higher rupture propensity of ACoA and MCA aneurysms may lead to rupture prior to screening, introducing detection bias in study populations.

The PHASES Score, proposed by Greving et al., identifies the diameter of UIAs as a predictive risk factor for rupture; UIA size also guides the decisions regarding the necessity of interventional operation. Furthermore, Schnell et al. (2014) identified how varying UIA sizes influence hemodynamics through differences in vortex and wall shear stress (WSS). High SR aneurysms exhibited multiple vortices and complex blood flow patterns, which significantly correlate with the likelihood of rupture. In IAs at vessel bifurcations, the NSI alone was linked to an increased risk of rupture. Miura et al. (2013) noted a significant relationship between AR, aneurysm neck width, and rupture risk. Conversely, Mader‐Sepahi et al. (2004) demonstrated that AR is more indicative of rupture risk than aneurysm size, whereas Baharoglu et al. (2010) confirmed that the incidence angle is an independent morphological predictor of IA rupture. They observed that higher incidence angles shift the recirculation zone towards the aneurysm apex, which is accompanied by higher inflow and higher maximal blood flow velocities, increased kinetic energy transitions to the apex, and greater wall shear stresses and its gradient (WSSG) at the apex. Additionally, greater bifurcation angles intensify the impact of blood flow on vessel walls at bifurcations, heightening rupture risk. Farnoush et al. (2013) investigated the effects of various bifurcation angles and branch artery diameters on aneurysms, finding that the intra‐aneurysm flow's kinetic energy attenuation is more responsive to changes in branch artery diameter than to bifurcation angle. Moreover, the ratio of arterial diameters before and after bifurcation inversely correlates with the rupture risk.

The supplementary analysis revealed that after stratification according to imaging modalities, no significant differences were observed in baseline characteristics, comorbidities, or parent artery distributions among groups. Most key morphological parameters demonstrated high consistency across imaging modalities. Notably, MRA tended to yield smaller measurements of aneurysm diameter compared to CTA and DSA, and it may underestimate aneurysm volume relative to DSA. However, CTA appeared to calculate lower NSI than DSA. Subsequent subgroup analyses of CTA and DSA cohorts (Table 7), while not reaching statistical significance, likely due to reduced sample sizes, demonstrated that the VM subgroup (*n* = 24) in CTA groups exhibited lower NSI than the migraine subgroup (*n* = 32). This finding indicates more irregular UIA morphology and a potentially higher rupture risk in the VM group, supporting the primary conclusion. In the DSA group, NSI values were nearly identical between the VM (*n* = 9) and migraine (*n* = 13) subgroups. This finding may be attributed to several factors: 1) the high resolution of DSA in detecting irregular UIA morphology could potentially dilute intergroup differences; 2) the DSA subgroup likely included more complex cases (e.g., larger aneurysms, as shown in Table 6), which may have confounded morphological comparisons; 3) the limited subgroup sample size may have reduced statistical power. Importantly, although the DSA results were not statistically significant, the CTA subgroup findings of lower NSI in VM patients consistently support our primary conclusion regarding rupture‐risk prediction.

**TABLE 7:** NSI differences between VM and migraine subgroups in CTA/DSA cohorts.

Items	CTA	DSA
Non‐spherical index		
VM	−30.74 (‐44.40, ‐18.77)	−21.40 (‐51.39, ‐17.29)
Migraine	−13.46 (‐15.34, ‐8.26)	−22.82 (‐33.50, ‐16.60)
*p*	0.112	0.616

Overall, these findings, based on morphological parameters and baseline clinical features, suggest that patients with VM and migraine are at an increased risk of IA rupture. Based on the evidence and hypotheses discussed earlier, it is recommended for VM and migraine patients to undergo imaging examinations to screen the UIA at the aforementioned locations. Once IA is confirmed, early endovascular treatment is recommended to prevent aSAH and to improve clinical outcomes. Long‐term follow‐up of migraine patients post‐intervention indicates that the frequency of headache episodes decreases regardless of the surgical method used, whether craniotomy or intervention (Valdueza et al. 2022). Therefore, despite the relatively low rupture risk, early intervention is advocated to also alleviate symptoms of dizziness and headache.

It can be inferred that patients with VM predominantly exhibit UIA in the C4, C5, and C7 segments of the internal carotid artery, whereas in migraine patients, they may be more commonly found in the C6 segment. The statistically significant distribution differences may reflect congenital abnormalities of arterial wall development in patients with VM and migraine or indicate specific locations of intracranial arteries that are particularly vulnerable under pathological conditions. We recommend that patients with VM and migraine undergo intracranial arterial imaging to exclude the potential presence of asymptomatic intracranial aneurysms in the previously mentioned locations. Furthermore, we propose a hemodynamics‐based hypothesis suggesting that the impact of UIA in these locations on hemodynamics, neural nuclei, and fiber bundle function may contribute significantly to the challenges in managing symptoms for patients with VM and migraine. Additionally, analysis of baseline clinical data and morphological parameters suggests an elevated risk of UIA rupture in patients with VM and migraine thus, early endovascular treatment is recommended to prevent aSAH and improve clinical outcomes.

This study faces several limitations: First, our cross‐sectional study reflects data from a specific period, limiting its ability to elucidate causal relationships between VM, migraine, and UIA, or to capture trends in aneurysm progression. As a result, it is important to recognize that our hypothesis represents only one interpretation based on the findings of this study, and it is neither comprehensive nor entirely objective. Direct causality and increased rupture risk in patients with VM, migraine, and UIA are still uncertain. Further validation through prospective studies, hemodynamic analyses, and animal experiments is necessary. Additionally, selection bias may have occurred. Second, despite morphology being an independent predictor of rupture (ROC‐AUC 0.693, *p* < 0.001) (Abboud et al. 2017), relying solely on morphological parameters to assess the risk of intracranial aneurysm rupture is incomplete. Third, a limitation stems from the heterogeneity of imaging modalities (CTA or MRA) across participants. While we implemented standardization protocols to ensure measurement comparability between modalities, future studies employing a uniform imaging protocol would enhance result consistency. Last, manual measurement is inherently subjective, and the precision of manual measurements remains difficult to ensure.

## Author Contributions

**Yaoheng Zhang**: conceptualization, investigation, writing – original draft, methodology, validation, visualization, software, resources, data curation, project administration, and funding acquisition. **Yi Ju**: conceptualization, methodology, supervision, project administration, writing – review and editing, and formal analysis. **Chunling Liu**: conceptualization, methodology, project administration, formal analysis, supervision, writing – review and editing. **Hui Li**: conceptualization, methodology, writing – review and editing, formal analysis, project administration, and supervision. **Yanlu Jia**: project administration, supervision, writing – review and editing, and methodology. **Shuning Sun**: supervision, formal analysis, project administration, writing – review and editing. **Haozhe Yin**: conceptualization, software, supervision, writing – review and editing. **Suisui Ma**: writing – review and editing, and supervision. **Wenbo Peng**: investigation and supervision.

## Disclosure

During the preparation of this work, the authors used ChatGPT 4.0 in order to improve readability and language. After using this technology, the authors reviewed and edited the content as needed and take full responsibility for the content of the publication.

## Ethics Statement

The studies involving human participants were reviewed and approved by the Ethics Committee of the Second Affiliated Hospital of Zhengzhou University. The participants provided their written informed consent to participate in this study.

## Conflicts of Interest

The authors declare no conflicts of interest.

## Peer Review

The peer review history for this article is available at https://publons.com/publon/10.1002/brb3.70782.

## Data Availability

The data that support the findings of this study are available from the corresponding author upon reasonable request.
